# Concealed Weapon Detection Using Thermal Cameras

**DOI:** 10.3390/jimaging11030072

**Published:** 2025-02-26

**Authors:** Juan D. Muñoz, Jesus Ruiz-Santaquiteria, Oscar Deniz, Gloria Bueno

**Affiliations:** VISILAB, Escuela Técnica Superior de Ingeniería Industrial, University of Castilla-La Mancha, 13071 Ciudad Real, Spain; juandaniel.munoz@uclm.es (J.D.M.); oscar.deniz@uclm.es (O.D.); gloria.bueno@uclm.es (G.B.)

**Keywords:** weapon detection, person detection, CCTV, handgun, thermal camera, Android application

## Abstract

In an era where security concerns are ever-increasing, the need for advanced technology to detect visible and concealed weapons has become critical. This paper introduces a novel two-stage method for concealed handgun detection, leveraging thermal imaging and deep learning, offering a potential real-world solution for law enforcement and surveillance applications. The approach first detects potential firearms at the frame level and subsequently verifies their association with a detected person, significantly reducing false positives and false negatives. Alarms are triggered only under specific conditions to ensure accurate and reliable detection, with precautionary alerts raised if no person is detected but a firearm is identified. Key contributions include a lightweight algorithm optimized for low-end embedded devices, making it suitable for wearable and mobile applications, and the creation of a tailored thermal dataset for controlled concealment scenarios. The system is implemented on a chest-worn Android smartphone with a miniature thermal camera, enabling hands-free operation. Experimental results validate the method’s effectiveness, achieving an mAP@50-95 of 64.52% on our dataset, improving state-of-the-art methods. By reducing false negatives and improving reliability, this study offers a scalable, practical solution for security applications.

## 1. Introduction

**Figure 1:**
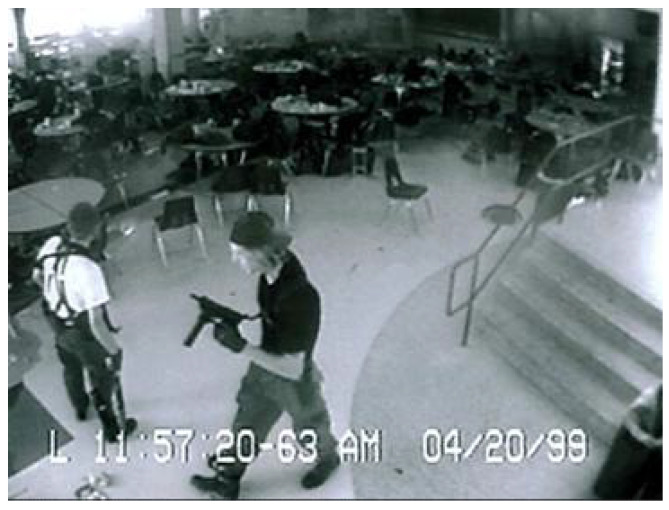
CCTV camera capturing the shooters of the Columbine High School tragedy that occurred on 20 April 1999.

In today’s increasingly complex security landscape, the need for robust and effective measures to detect weapons has never been more critical. As threats to public safety evolve (whether from terrorism, organized crime, or mass shootings, as shown in Figure 1, the shooting that took place on 20 April 1999 at Columbine High School [1]), security professionals face the daunting task of preventing potential disasters while balancing efficiency and practicality.

The rising sophistication of weapon concealment tactics, combined with the increasing ease of access to dangerous weapons, calls for a new wave of detection technologies that can adapt to these emerging threats. Traditional methods, such as metal detectors and X-ray scanners, are often limited by their scope and are not always effective in detecting non-metallic or cleverly disguised weapons. This has led to a growing demand for advanced systems that can offer higher accuracy, faster response times, and greater adaptability to different environments and threat levels.

To address these challenges, this paper delves into the synergistic convergence of two powerful technologies: thermal imaging and deep learning. Thermal imaging operates passively by capturing infrared radiation naturally emitted by objects, ensuring safety for both subjects and operators. Its ability to detect temperature variations allows thermal cameras to identify concealed firearms, which exhibit distinct thermal signatures compared to the human body or clothing. Deep learning, on the other hand, offers the ability to analyze large datasets and automatically recognize complex patterns, reducing false positives (and false negatives, which is key in this field) and improving detection accuracy. The combination of these technologies provides a powerful solution that addresses the limitations of traditional methods.

**Figure 2:**
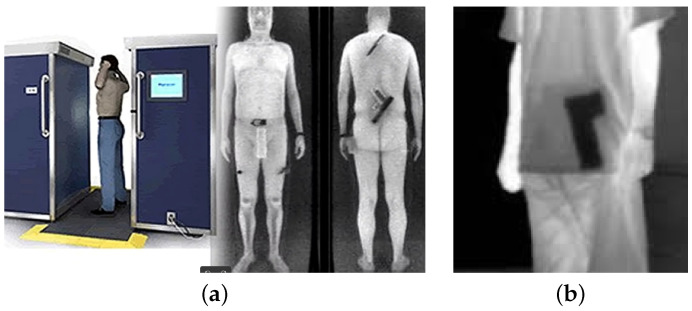
(**a**) Example of an X-ray scanner used to detect concealed objects on a person [2]. (**b**) Example of an infrared camera used to detect concealed objects on a person [3].

Thermal imaging is particularly advantageous due to its non-intrusive nature, preserving privacy better than methods that generate detailed anatomical outlines (see Figure 2). It also operates effectively in low visibility conditions, including total darkness, making it suitable for diverse security scenarios. The initial investment may be significant, but its durability and low operational costs make it a cost-effective choice over time, particularly for applications requiring frequent and rapid screening. By augmenting thermal imaging with deep learning, systems can enhance weapon detection by quickly analyzing heat signatures with high precision while adapting to new threats.

This combination represents a cutting-edge approach to concealed weapon detection that holds the potential to revolutionize security measures across a variety of sectors, including airports, public transportation hubs, government buildings, and other high-security areas. Our method is based on two detection stages: frame-level handgun detection and frame-level person detection. First, the handgun detector decides whether there is a gun in the processed frame or not, discarding it if nothing is detected. Nevertheless, if a gun is detected, then a person detector is applied and it is checked if the previously detected gun is contained in the person’s area. If no person is detected, an alarm is triggered either way, just in case; if a person is detected and the gun is contained in that area, an alarm is triggered; if a person is detected and the gun is not contained in that area, the alarm is not triggered, as the gun detection is not considered to be legit.

## 2. Previous Work

Thermal imaging technology has played a significant role in surveillance and security systems, particularly for detecting concealed threats. The following studies highlight relevant advancements in the field and provide context for the contributions of the present work.

In 2010, Kastek et al. [4] explored the use of infrared cameras for sniper detection, demonstrating the capability of thermal imaging to identify muzzle flashes and camouflaged snipers. While this application differs from concealed weapon detection under clothing, it highlighted the potential of infrared technology for identifying threats in visually obscured scenarios. Their work also revealed the limitations of thermal imaging at long distances, emphasizing the need for high-performance sensors, which remains a consideration in modern applications. The focus of this study on sensor selection and operational challenges influenced our approach to optimize detection performance for shorter distances using compact and affordable thermal cameras.

Building on this foundation, Jedrasiak et al. [5] proposed a prototype for concealed weapon detection (CWD) in 2012 that integrated infrared and visual cameras with a fast image fusion algorithm. Their work demonstrated the feasibility of combining thermal and visible-light imaging to enhance detection performance. However, the system faced limitations in terms of scalability, real-time processing, and deployment practicality due to its reliance on custom hardware. This highlights the importance of designing systems compatible with readily available components, which we address by leveraging off-the-shelf thermal cameras and Android-based smartphones in our work.

In 2017, Nashwan et al. [3] introduced a hybrid algorithm combining discrete wavelet transform and shape-matching-based Support Vector Machine (SVM) classification [6]. Their approach improved image quality through sensor fusion, aiding in concealed weapon detection from a standoff distance. However, the reliance on handcrafted features and limited datasets constrained the scalability and adaptability of the method. In contrast, our study employs deep learning-based approaches capable of end-to-end learning, reducing dependence on manual feature engineering and improving performance on diverse datasets.

More recently, Gaurav et al. [7] in 2019 applied Faster Region-based Convolutional Neural Network (Faster R-CNN) [8] for concealed weapon detection using thermal and HD cameras integrated with Raspberry Pi and Intel Movidius accelerators. While their system achieved a commendable 93.6% accuracy, its reliance on specific hardware accelerators and limited dataset diversity posed challenges for broader deployment. Our work addresses these limitations by focusing on lightweight neural network architectures that operate efficiently on low-power, widely available embedded devices.

In 2022, Hema and Subramanian [9] proposed an infrared-imaging-enabled drone for weapon detection, emphasizing autonomous surveillance. Despite its potential, the approach was hindered by distance limitations, making it unsuitable for scenarios requiring close-range detection. Additionally, the cost and maintenance of autonomous drone systems limit their practicality. In contrast, our work prioritizes compact, wearable solutions optimized for close-range applications, aligning better with real-world operational requirements.

More recently, in 2023, Veranyurt et al. [10] proposed a deep learning-based system for detecting and locating concealed pistols in thermal images for real-time surveillance, combining two models: one for classification and another for detection. Using a self-created dataset of 600 images [11], along with the Trimodal [12] and Linköping Thermal InfraRed (LTIR) [13] datasets, the method preprocesses thermal images to enhance sharpness and contrast before classifying the presence of a weapon. If detected, the system generates a bounding box to locate the pistol and uses a security counter to trigger alarms after consecutive detections. The best results were achieved with a fine-tuned Visual Geometry Group 19 (VGG19) model [14] for classification (85% accuracy, 85% precision, F1-score of 0.84) and a fine-tuned You Only Look Once version 3 (YOLOv3) model [15] for detection (a mean Average Precision, or mAP, of 95% at 10 Frames Per Second (FPS) and a 92% mAP for classification). Despite promising results, the authors suggested improvements such as adopting newer architectures, increasing FPS by using enhanced hardware configurations to avoid missed detections, expanding datasets for varied conditions, and considering multi-person scenarios for greater system robustness.

With that all said, the present paper paper makes several significant contributions to the field of concealed weapon detection, specifically focusing on the use of thermal imaging technology. Our main contribution is the algorithm presented, as we propose a straightforward yet effective method for detecting concealed weapons. Our approach is designed to be computationally efficient, allowing it to be implemented on low-end embedded devices, which are increasingly prevalent in mobile security applications. This is particularly important because security personnel often require portable solutions that can function in various environments without relying on high-power computing resources. By optimizing the algorithm for performance on less powerful hardware, we enhance accessibility and practicality for real-world applications. Along with that main objective, we also introduce a thermal image dataset tailored explicitly for concealed handgun detection at relatively short distances. Our dataset includes a diverse range of samples that represent different types of clothing and concealment techniques, making it a valuable resource for training and testing detection algorithms. Finally, we implement and evaluate our detection method using a chest-worn Android smartphone connected to a miniature thermal camera. This setup allows for hands-free operation, enabling security personnel to maintain situational awareness while monitoring concealed weapons. As far as the authors are aware, this is the first study to address concealed handgun detection using this specific combination of a self-made thermal imaging dataset, efficient algorithm, and wearable technology. Our results indicate promising detection performance, demonstrating that the method can effectively identify concealed weapons while maintaining a manageable computational burden. This balance between detection accuracy and processing efficiency opens the door to potential real-world applications, such as airport security, public event monitoring, and law enforcement operations.

## 3. Materials and Methods

### 3.1. Datasets

Before delving into the explanation of the proposed method, it is important to describe how the method is going to be tested. In this case, along with an Android app implementation, results have been extracted from testing two different datasets: the “Concealed Pistol Detection Dataset” [11], which is an extract from study [10], consisting of 358 non-annotated images (since concealed objects were not annotated in the available dataset, it was necessary to manually annotate all of the images containing concealed handguns within them. This dataset contains only one type of concealed object (thus one class): “Handgun”), and the “UCLM Thermal Imaging Dataset”, by Muñoz et al. [16], which is a dataset introduced in this paper consisting of 102 annotated thermal videos, categorized into four classes: “Handgun”, “Smartphone”, “Keys” and “Person”. The videos were collected using the TOPDON TC001 thermal camera, produced by TOPDON Technologies [17].

For both datasets, the ground truth needed to be generated (in this paper, for evaluation purposes, only the “Handgun” class annotations were considered). This was carried out by an expert, who manually annotated each frame using two tools: for the “Concealed Pistol Detection Dataset”, the Computer Vision Annotation Tool (CVAT) [18], and for the “UCLM Thermal Imaging Dataset” ground truth, the Image Labeler from *MATLAB* [19] (the version of *MATLAB* used was the R2023a, the 9.14). The criterion followed by the expert was to visually identify, by inspecting each image, whether the individual was carrying a concealed object in a pocket or under other garments or not. This visual inspection is feasible due to the thermal camera’s temperature sensor, which highlights concealed objects like weapons made of metal by generating distinct shapes caused by their lower temperature. These shapes enable the identification of hidden objects and their corresponding classes. Although the images were not categorized by difficulty of identification, some cases are inherently easier to label than others. For instance, if the object of interest (in this case, the weapon) has recently come into contact with the body, there may not be enough time for the garment to cool as a result of the concealed object’s temperature, reducing the visible temperature contrast. Also, if a concealed handgun has been in contact with the body for an extended period, it may warm up, reducing the thermal contrast required for reliable detection. Another challenging scenario arises when the weapon is concealed beneath thick or insulating clothing, which prevents the weapon from being visualized. Implementing data augmentation techniques in the dataset could help increase the variety of images and improve the identification of concealed objects across a wider range of scenarios.

**Figure 3:**
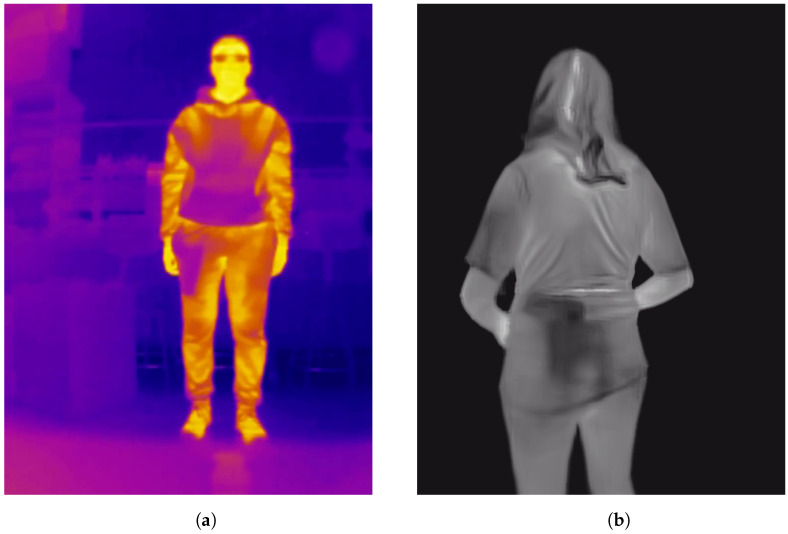
(**a**) Sample image of the “UCLM Thermal Imaging Dataset” where there is a concealed handgun. (**b**) Sample image of the “Concealed Pistol Detection Dataset” where there is a concealed handgun.

Images of both datasets are shown in Figure 3. Moreover, to demonstrate that the results obtained improve generally when applying our proposed method and not only in specific cases, for each dataset five experimental runs per method were performed. Each run varies based on the dataset variant selected. In the case of the “Concealed Pistol Detection Dataset”, due to the limited number of images, all images were selected, and for each run, a random split of the dataset into training/validation/test sets was carried out, always with a 60/10/30 ratio, respectively. For the “UCLM Thermal Imaging Dataset”, it was first reduced by selecting one out of every five images, resulting in a total of 2402. This was carried out to avoid overfitting due to the high similarity of consecutive images. With these 2402 images, five runs were carried out, randomly splitting them into training/validation/test sets, maintaining a 60/10/30 ratio for each set, respectively. Note that although we reduced our dataset to obtain metrics to avoid overfitting, the dataset was created with a specific scenario in mind: a police officer wearing a mobile phone on his chest connected to a thermal camera that, via a real-time application (described in Section 4.5), receives an alert if a concealed weapon is detected on the individual in front of the camera.

### 3.2. Proposed Method

**Figure 4:**
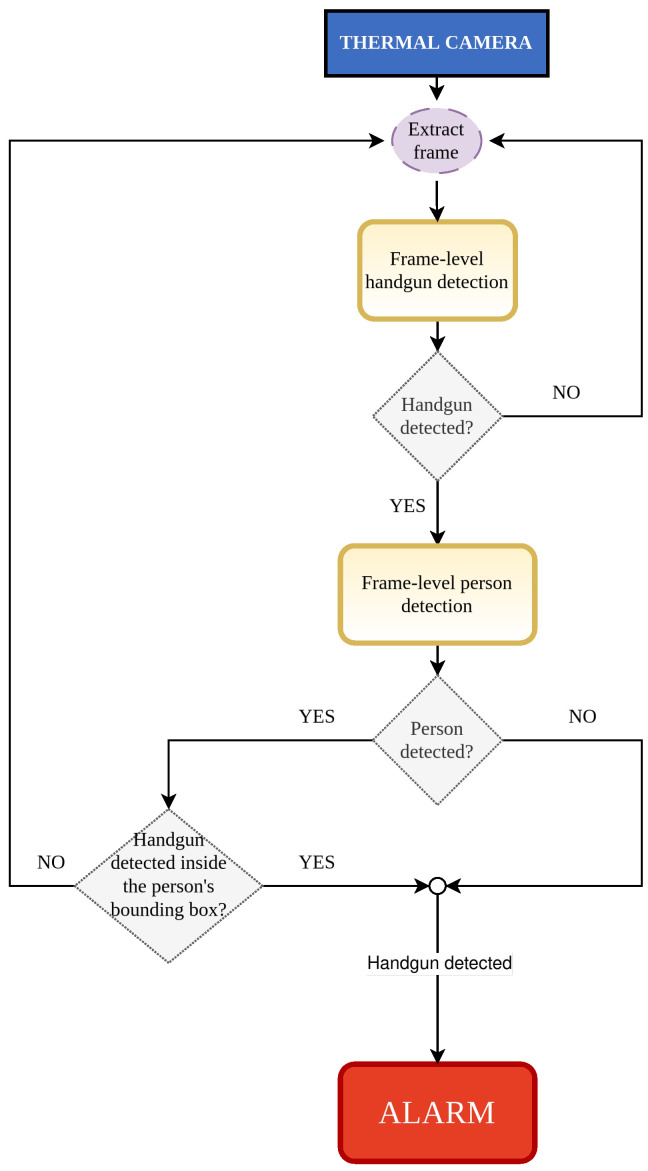
Outline of the proposed method. Key steps are detailed. These include the initial handgun detection at the frame level, followed by the use of a person detector to define the area of interest.

The method proposed is described in Figure 4. This method aims to improve existing methods by reducing false positives (unreal guns detected as real) along with reducing false negatives (missed real guns). In most cases, a firearm is carried by an individual; therefore, considering only handgun detections within the region corresponding to a person identified in the image helps mitigate false positives outside the area of interest. Moreover, in instances where a handgun is detected during the initial step of the method but no person is identified, an alarm is triggered as a precautionary measure to account for potential failures in the person detection process. A more detailed explanation is provided below.

**Figure 5:**
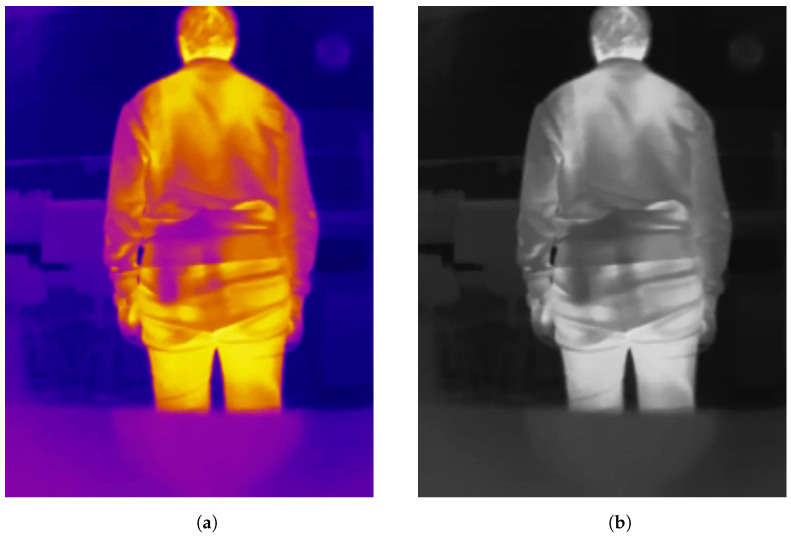
(**a**) Example of an original frame (hot colormap) from the “UCLM Thermal Imaging Dataset”. (**b**) Frame of the “UCLM Thermal Imaging Dataset” after being converted from *hot* colormap to grayscale.

**Figure 6:**
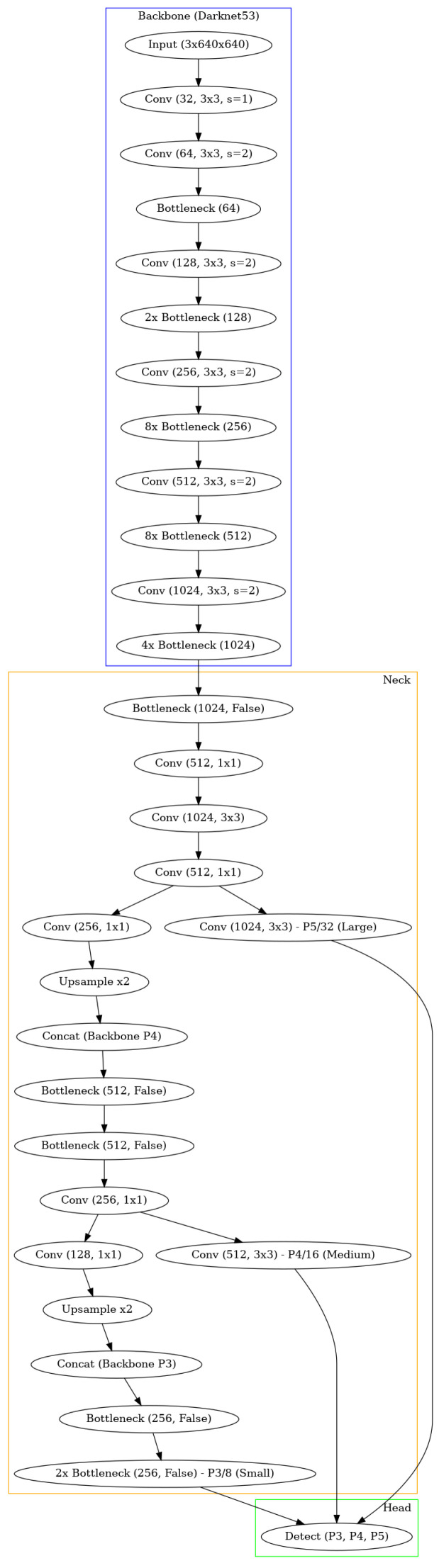
Simplified architecture of YOLOv3u obtained using *Graphviz* [25] in its 0.20.3 version. The backbone (Darknet53 [24]) is responsible for feature extraction and consists of a series of convolutional (abbreviated as “Conv” in the figure) layers and bottlenecks, which are blocks designed to learn and capture abstract feature representations of the input data while reducing computational complexity. The neck includes upsampling layers (which double the input dimensions without weights) and concatenation operations that fuse multi-scale features to improve the model’s detection capabilities. The head is responsible for generating detections at three different scales (P3, P4, P5). Moreover, in the figure, “Concat” refers to concatenation.

**Figure 7:**
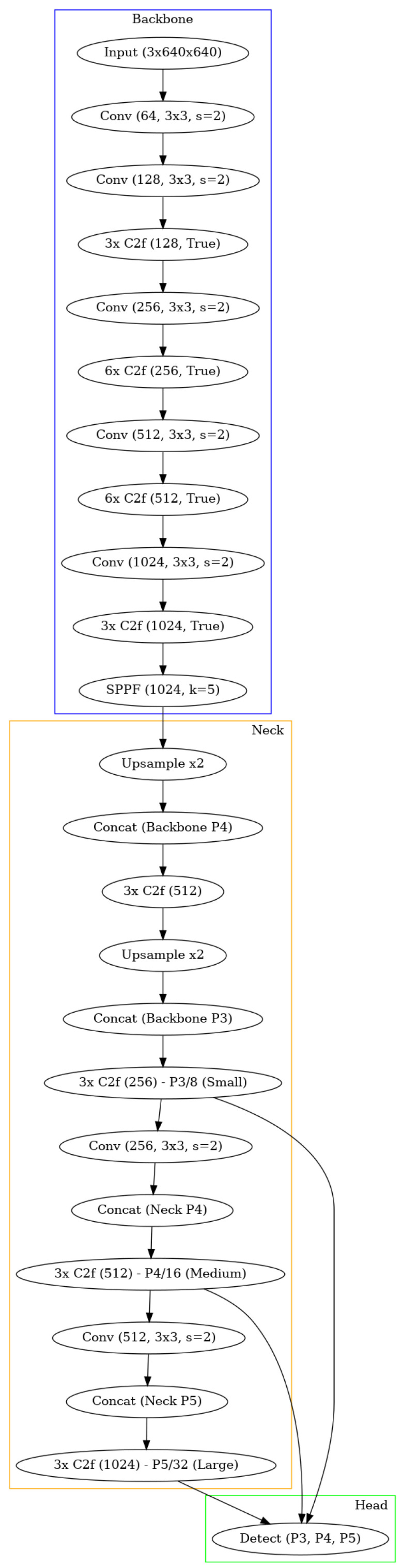
Simplified architecture of YOLOv8x obtained using *Graphviz* [25]. The backbone is responsible for feature extraction and consists of a series of convolutional (abbreviated as “Conv” in the figure) layers and C2f blocks. Each C2f block involves a convolutional layer, where the resulting feature map is split. One part goes through a Bottleneck block, and the other is directly passed to a Concat block, with the two outputs being combined before a final convolution. The backbone also includes an Spatial Pyramid Pooling-Fast (SPPF) block, which applies pooling operations at multiple scales to capture multi-scale information and handle images of different resolutions more effectively. The neck includes upsampling layers (which double the input dimensions without weights) and concatenation operations to fuse multi-scale features. Finally, the head generates detections at three different scales (P3, P4, P5). Moreover, in the figure, “Concat” refers to concatenation.

**Preprocessing.** To initiate the process, a frame from the thermal camera feed is first extracted. We tested our method in two configurations: one using the described datasets and another by integrating it into an Android app. For the datasets, images were converted to grayscale. Aside from this conversion, no additional preprocessing, such as contrast adjustment, was applied. For the Android app implementation, the camera outputs grayscale images directly, making further preprocessing unnecessary. Taking heed of the conversion of the images of the datasets to grayscale (the only preprocessing operation carried out), this was only needed in the case of the “UCLM Thermal Imaging Dataset”, as the “Concealed Pistol Detection Dataset” images were already in grayscale. The conversion was performed in Python in its 3.10 version [20] with the *OpenCV* library [21]. After loading the original images, which had a *hot* colormap applied [22] to them, we used the *cv2.COLOR_BGR2GRAY* option to convert them into grayscale. *COLOR_BGR2GRAY* color mode applies the Equation (1) to convert from any three-channel image to a one-channel image. See Figure 5 as an example of the conversion operation of a frame from the “UCLM Thermal Imaging Dataset”.
  (1) Y=0.299·R+0.587·G+0.114·B
**Handgun detection.** Once the image is obtained, we perform frame-level handgun detection using a trained object detection model. This step confirms the presence of a concealed handgun within the frame. If a handgun is detected, the bounding box coordinates of each detected concealed handgun are recorded for further analysis. If no handgun is detected, the current frame is discarded, and the next frame is processed.
The chosen architecture to perform this task was a YOLOv3 [15], more specifically the YOLOv3u version (YOLOv3u architecture in Figure 6), which is an updated version of YOLOv3-Ultralytics that incorporates the anchor-free, objectness-free split head used in YOLOv8 [23] models (YOLOv8 version *x* architecture in Figure 7). YOLOv3u maintains the same backbone (*Darknet53* [24]) and neck architecture as YOLOv3 but with the updated detection head from YOLOv8.

**Figure 8:**
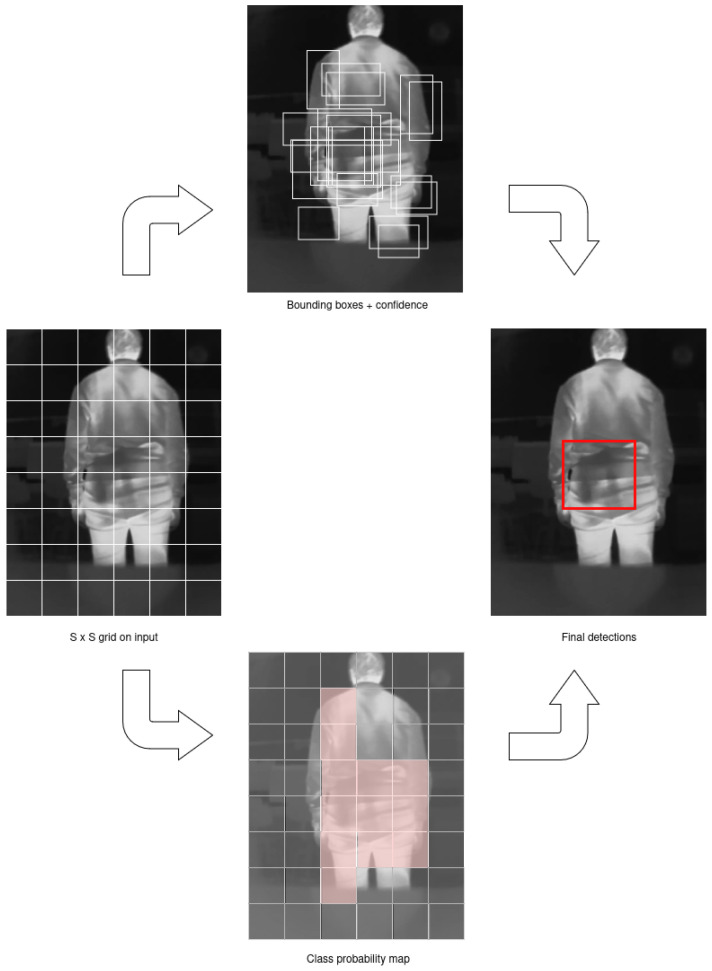
The process followed by YOLO to classify and localize objects within an image. The model divides the input image into a grid and assigns each grid cell the responsibility of predicting objects that fall within its boundaries. Each grid cell predicts a fixed number of bounding boxes along with class probabilities and objectness scores. The network then processes these predictions through the model’s layers, producing the final object classifications and accurate localization through bounding-box coordinates for each detected object in the image.

We chose to use a YOLO mainly because of its ability to make predictions about object locations and classes quickly in a single pass over the image, unlike other object detection methods that require multiple stages or “passes” of the image. YOLO divides the input image into a grid of *S* × *S* cells. Each cell is responsible for predicting whether an object exists in that region of the image or not. As newer versions were developed, mechanisms were improved to detect multiple objects per cell and better handle small objects. Each cell in the grid generates several bounding boxes, which are rectangles that could potentially contain an object. Each bounding box includes the coordinates (x,y) for the center of the object; the width and height of the box; a “confidence score”, which represents the probability that there is actually an object in that box. For each bounding box, YOLO also predicts a set of classes (e.g., “person”, “dog”, “car”) with associated probabilities. Finally, YOLO uses a method called Non-Maximum Suppression (NMS) [26] to reduce redundant predictions. The general process followed by a YOLO model is shown in Figure 8.

**Person detection.** Upon confirming the presence of a concealed handgun within the frame, the person detection process is initiated. The person detection model identifies individuals within the image and selects the bounding box the model is most confident in (considering only one person for simplicity). Using the coordinates of the detected handgun(s) from the prior step, the system checks whether the detected handgun is located within the bounding box of the identified person. To ensure a more reliable assessment and reduce the risk of false positives, the sides of the person’s bounding box are initially expanded by 15%, allowing a margin for detection. Following this adjustment, a straightforward comparison is made between the coordinates to verify whether a handgun lies within the person’s bounding box. However, in the absence of any detected persons, the firearm with the highest confidence score (reflecting the model’s highest level of certainty) shall still be considered valid, provided that the handgun detector has identified at least one firearm.
In this case, we used a pretrained YOLOv8, specifically the YOLOv8x version, which is the best Ultralytics version; it is also the one that requires the most capacity but works better than the others. The model was pretrained to detect 80 classes, but only the “Person” class was considered to carry out the experiments.
**Alarm.** Finally, if at least one handgun detection meets the criteria (either located within the expanded bounds of a person’s bounding box or detected independently of a person), the system triggers an alarm.

### 3.3. Setup

In order to carry out the proposed method, some aspects need to be considered.

**Figure 9:**
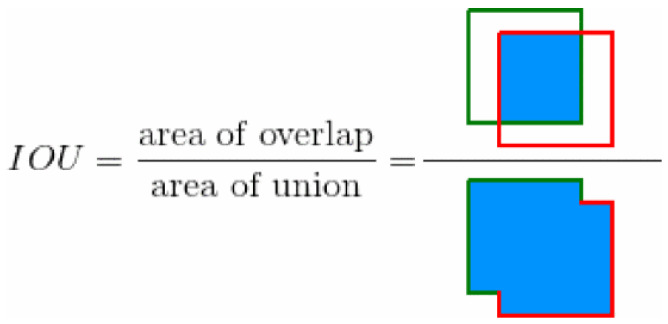
Intersection over Union (IoU) [28]. The figure graphically illustrates this concept with two squares, representing the predicted and ground truth bounding boxes, highlighting both the overlapping region and the union of the two areas to show how IoU is calculated.

**Experimental setup**. Training and testing were performed on an ${\mathrm{Intel}}^{®}$ Xeon(R) CPU E5-2620 (produced by Intel) computer with a NVIDIA Quadro P4000 GPU (produced by NVIDIA). We chose to use the TOPDON TC001 thermal camera [17], which was selected due to its small size and reduced cost. The camera was directly connected to a HUAWEI P30 Lite Android smartphone (produced by HUAWEI) and videos for the “UCLM Thermal Imaging Dataset” were collected (later, images were extracted from these) using the *TC001*’s official Android app. Later, the same thermal camera and the same smartphone were used in order to test the Android app.
**Metrics**. To evaluate the methods, considering that they are primarily detectors, the mean Average Precision (mAP) [27] has been extracted, which is a widely used metric to evaluate the performance of object detection models, such as YOLO or Faster R-CNN. To fully understand mAP, we need to break down the fundamental concepts it builds upon [28]: true positives (TPs), false positives (FPs), false negatives (FNs), true negatives (TNs), precision, recall, Intersection over Union (IoU), and finally, Average Precision (AP) and mAP. In object detection, predictions are evaluated based on their alignment with ground truth (information that is known to be real or true) objects. This is done using the following definitions:

- **True Positive (TP).** A detection is considered a true positive when the predicted bounding box correctly identifies an object and sufficiently overlaps with the ground truth bounding box. Before explaining the next concepts, it is key to understand what “overlapping” means in this context, and that is the Intersection over Union (IoU). In the object detection scope, the IoU measures the overlapping area between the predicted bounding box *B_p* and the ground truth bounding box *B_gt* divided by the area of union between them (see Equation (2)). By comparing the IoU with a given threshold *t*, we can classify a detection as being correct or incorrect. If IoU≥t then the detection is considered as correct. If IoU<t the detection is considered as incorrect. For a better understanding, the IoU concept is graphically described in Figure 9.
  (2) $$IoU=\frac{area(B_{p}\cap B_{gt})}{area(B_{p}\cup B_{gt})}$$
- **False Positive (FP).** A detection is considered a false positive when the model predicts a bounding box that does not correspond to any actual object, or when the bounding box overlaps insufficiently with a ground truth box.
- **False Negative (FN).** A false negative occurs when the model fails to detect an object that is present in the image.
- **True Negative (TN).** This term is less relevant in object detection as it refers to correctly identifying the absence of objects, which is not explicitly evaluated.

Using the above definitions, we can derive two core evaluation metrics: precision and recall. Precision measures the proportion of correctly predicted objects (true positives) relative to the total number of predictions made (true positives + false positives). It is calculated as shown in Equation (3). A high precision indicates that the model makes few incorrect predictions (false positives). Recall measures the proportion of real objects that were successfully detected by the model (true positives) relative to the total number of actual objects (true positives + false negatives). It is calculated as shown in Equation (4). A high recall means that the model detects most of the objects present in the image.

(3) $$Precision=\frac{TP}{TP+FP}$$

(4) $$Recall=\frac{TP}{TP+FN}$$

To evaluate a model across different thresholds for classification confidence, we calculate precision and recall for various confidence levels and plot a precision–recall (*PR*) curve. The *PR* curve shows how precision and recall trade off as the model’s confidence threshold changes. The AP summarizes the *PR* curve into a single number by calculating the area under the curve (*AUC*). It is computed as shown in Equation (5).

(5) $$AP=\int_{0}^{1}Precision\left(R\right)dR$$

where *R* represents recall.

Finally, the mAP extends the concept of AP to evaluate the model’s performance across all object classes. For a model that detects *N* object classes, mAP is calculated as in Equation (6).

(6) $$mAP=\frac{1}{N}\sum_{i=1}^{N}AP_{i}$$

where *AP_i* is the average precision for class *i*. To account for varying detection strictness, mAP can be evaluated at different IoU thresholds, i.e., the overlapping concept explained above (e.g., 0.5, 0.75, or averaged over a range like [0.5:0.95]). In this paper, the mAP has been extracted in the range [0.5:0.95].

In this paper, the metrics were extracted by considering the results from five runs of each dataset per method (i.e., one mAP@50-95 value per run). The final result for each method is reported as the average of these five runs, along with the corresponding standard deviation.

When extracting the metrics, it is necessary to assign a confidence threshold to the detectors (handgun and person). This confidence threshold is independent of the IoU threshold, as it is not used to compare predictions with ground truth. The confidence threshold represents the probability score that the detector assigns to the presence of an object in a given bounding box. A lower confidence threshold is less restrictive, resulting in the model detecting more objects in a frame, albeit at the cost of more false positives. Conversely, a higher confidence threshold is more restrictive, yielding fewer detections but with greater precision and fewer false positives.

To calculate the mAP metric, we opted for a low confidence threshold for the handgun detector (set at 0.0001) to include all potential detections. Selecting a low detector threshold is essential for accurately extracting the mAP metric. It ensures that all potential detections, including those with low confidence, are considered during evaluation, allowing for a complete analysis of the precision–recall trade-off across all confidence levels. Using a higher threshold would exclude many valid but low-confidence detections, leading to incomplete precision–recall curves and an inaccurate assessment of model performance. By adopting a low threshold, the evaluation better reflects the model’s comprehensive detection capabilities. Similarly, for the person detector, the confidence threshold was also set to 0.0001. However, in this case, only the highest-confidence detection per frame was considered, as the goal was not to maximize the number of detected individuals (the method is proposed to detect guns, not people, and thus metrics refer to final gun detections).

Finally, it should be noted that the use of low confidence thresholds is strictly for metric extraction purposes. In a real-time system, these thresholds would be set to higher values to reduce the occurrence of false positives.

**Detection Task.** Once the datasets, the proposed method, the experimental setup, and the evaluation metrics are defined, the question arises regarding the objective to be achieved. On one hand, the proposed method has been developed with the aim of minimizing false positives, as weapons are encapsulated within the person. On the other hand, the primary and most critical objective is to ensure that no detection is missed, as detecting hidden weapons is of utmost importance. Therefore, a balance is sought between avoiding missed detections while filtering out unnecessary ones. For this reason, in cases where no person is detected, the method triggers an alarm regardless, provided a weapon has been detected at the frame level.
**Training Process**. Once the architectures and the metrics used have been explained, before the testing process they had to be trained. The chosen YOLOv8x to perform the person detection task was pretrained, so only the YOLOv3u needed to be trained. It is important to note that, when training a YOLO model, it internally resizes image data to a range [0,1] by dividing each pixel value by 255 to work more efficiently. Moreover, a default data augmentation is performed during the YOLO training, which helps the architecture in detecting better in difficult situations. This default data augmentation consists of the following: color augmentations involve random adjustments to hue (±1.5%), saturation (up to ±70%), and brightness (up to ±40%); geometric transformations include random translation (±10%), scaling (up to ±50%), and horizontal flipping with a 50% probability; mosaic augmentation, a key feature in YOLO models that combines four images into one, is enabled; random erasing with a 40% probability is applied to improve robustness, and auto-augment with the *RandAugment* policy introduces further random transformations; and the crop fraction is set to 1.0, ensuring no cropping is applied.

Now, delving into the datasets and hyperparameter details, see Table 1 for more information. Furthermore, in order to split the datasets into train/val/test, stratified shuffling has been used (60%/10%/30%, respectively).

Note that, within each dataset, each run has been trained using the same hyperparameters. In addition to what is shown in Table 1, the YOLOv3u was trained chosing, for both datasets, the same number of epochs (15), the same input size for the images (128×128), the same optimizer (AdamW with a 0.9 momentum) and the same initial learning rate (0.002).

The metrics reported for the trained models were obtained using the validation option in the Ultralytics framework, specifying the path to the test dataset. These metrics represent the average performance over five runs. For the “UCLM Thermal Imaging Dataset”, the model achieved a precision and recall of 94.58%, with an mAP@50 (IoU = 0.50) of 95.08% and an mAP@50-95 (IoU from 0.50 to 0.95) of 65.44%. On the “Concealed Pistol Detection Dataset”, the model recorded a precision of 50.62%, recall of 70.48%, an mAP@50 of 55.40%, and an mAP@50-95 of 22.35%. These metrics help determine whether further training is required. The results are presented separately here, as they reflect the performance of the trained models in detecting the specific classes they were trained on. They are independent of the method’s results and are not included within them.

## 4. Results

### 4.1. Complete Method Results

Now that the architecture has been carefully trained, not allowing overfitting to happen, a Python code is developed (not publicly available) and executed to test the complete method, which is described in Figure 4. In order to obtain the results, a ground truth for each run has been created (a JavaScript Object Notation file, more commonly known as JSON, in COCO standard format). Then, the complete method code is executed, storing the detections of the test set in another JSON file. Finally, the results for each run (object detection metrics, mAP varying the Intersection Over Union, or IoU, from 0.50 to 0.95) are obtained by comparing the JSON files. As previously explained, all handgun detections have been considered and the results have been extracted setting a 0.0001 confidence threshold for both architectures. Nevertheless, results considering only the best handgun detection (meaning only including the bounding box with the greatest score among all) have also been included to add more information. Apart from this, JSON files only contain “Handgun” detections. This means that when showing the mAP metric, it is equal to the AP value, as there is only one final class. Results are shown in Table 2. Moreover, an ablation experiment is shown in Section 4.2 to realize the impact of the person detector in the proposed method.

First, note that in Table 2, when considering only the best score detection per frame, the results are worse. This is due to the fact that by selecting only the highest-scoring detection per frame, you are potentially discarding other valid detections that may have a lower score but are still accurate. In some cases, the model may produce multiple detections for the same object with varying confidence levels, and by only considering the highest confidence, you may miss out on the overall accuracy of the model. Additionally, this approach may reduce the diversity of detections across frames, which could impact the mAP@50-95 performance, particularly in challenging detection scenarios where multiple objects are close together or occluded.

**Figure 10:**
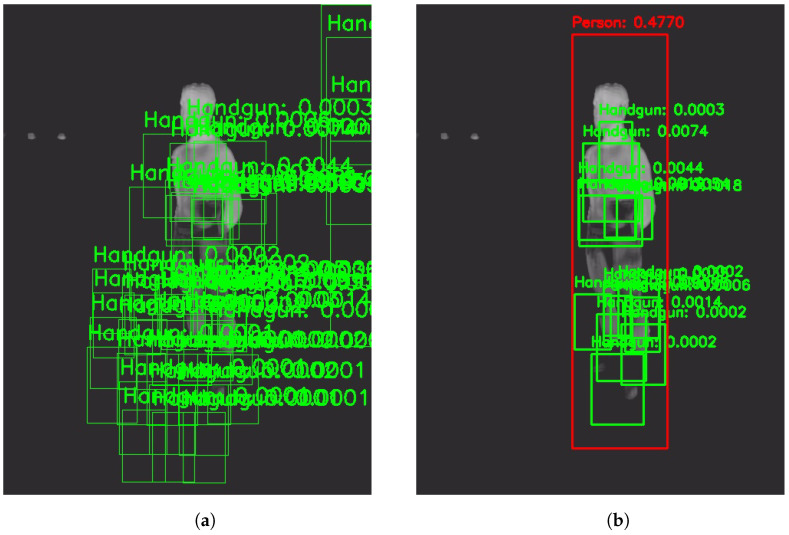
(**a**) Result with the method proposed in [10]. (**b**) Same image, result with the proposed method. “Concealed Pistol Detection Dataset”. All detections considered. Models’ min confidence value: 0.0001.

To compare with other state-of-the-art methods, we replicated [10] and tested it with both datasets. Results show that our method consistently improves on ≈1% all metrics. For instance, an mAP@50-95 of 27.18% is achieved when testing [11] considering all handgun detections per frame. In this way, our method achieves better our goals because mAP@50-95 measures the balance between precision (reducing false positives) and recall (reducing false negatives) across a range of IoU thresholds (0.5 to 0.95). Then, a higher mAP indicates that the our method achieves a more effective trade-off between these two goals, consistently performing better at detecting true positives while avoiding false detections (see Figure 10). The improvement, however, is not great. This is primarily because of the use of the same trained handgun detector (YOLOv3u) because we wanted to demonstrate that the improvement comes from the method itself and not the architecture selected. Additionally, our dataset, the “UCLM Thermal Imaging Dataset”, has limitations. It includes only a few scenarios with minimal variation, such as consistent lighting conditions, the same individual, and the same room throughout. This is a first approach and this data could be improved. Nonetheless, this dataset was created with the intention of helping a police officer to detect hidden weapons when facing a person, so the situations do not vary too much (there is no movement in the scene and the person will always face or have their back to the officer).

Note also that when considering the “Concealed Pistol Detection Dataset”, results are worse in general due to the lack of images of the dataset, along with a lot of variability among them, thus leading to a worse training of the model and a worse performance. Nevertheless, by testing on a “worse” dataset, we aim to demonstrate that our method improves results, regardless of the data quality. The datasets themselves are not being compared, nor should they be, as the goal is not to compare datasets but to show how our method improves results in comparison to existing methods.

**Figure 11:**
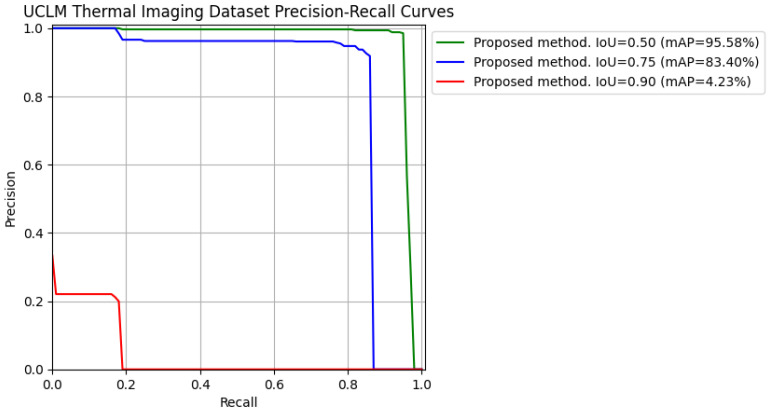
Precision–recall curves for IoU = [0.50, 0.75, 0.90] obtained from testing the proposed method on the “UCLM Thermal Imaging Dataset”.

**Figure 12:**
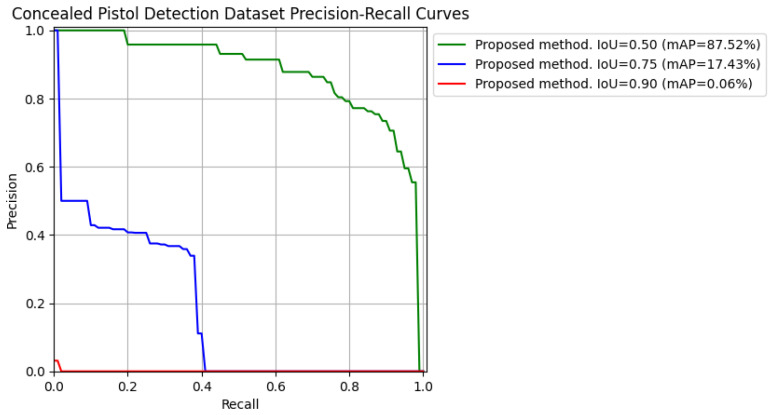
Precision–recall curves for IoU = [0.50, 0.75, 0.90] obtained from testing the proposed method on the “Concealed Pistol Detection Dataset”.

To see it clearer and graphically, precision–recall curves (varying IoU) for the first run of each dataset tested with the proposed method have been extracted so that it is easier to understand the mAP results. See Figure 11 and Figure 12.

### 4.2. Ablation Experiment

In order to realize how important is to have the person detector in terms of what we want to achieve, we can extract mAP metrics by removing this architecture, thus testing the model only with the handgun detector. Results are shown in Table 3.

Comparing with results shown in Table 2, these in Table 3 are a bit lower, meaning that the person detector influences the discarding of many false positives, which is one of the main aims of the proposed method.

### 4.3. Classification Metrics

Also, classification metrics of the proposed method have been extracted varying the confidence of the detectors (these are not the IoU thresholds but the architecture’s threshold to consider detections). These metrics (shown values are the mean of the runs) represent when the proposed method determines “Handgun” or not when there is a gun within the image (true positive or false negative, respectively). They also represent when the method detects “Handgun” or not when there is actually not a gun in the image (false positive or true negative, respectively).

These are not detection metrics. This means that if a gun is present and “Handgun” is detected, it will be counted as a true positive. However, this is the case even if the bounding box of the detection does not align with the ground truth annotation. Also, to stick as much as possible to a real situation, only the detection with the highest score per frame has been considered. All detections are not needed because mAP is not being extracted. See Table 4 and Table 5. To facilitate a comparison with other state-of-the-art methods, classification metrics were also obtained for the approach presented in [10]. The results indicated a higher occurrence of both false positives and false negatives in that method, thereby demonstrating the superior performance of our proposed approach in achieving the intended objectives.

### 4.4. Comparison with State-of-the-Art Method

With all metrics calculated and having shown some metrics resulting from testing with the method described in [10], an exhaustive comparison between this and our method is shown in Table 6, Table 7 and Table 8. This supplementary section aims to demonstrate that our method outperforms existing state-of-the-art approaches in achieving our primary objective: minimizing false positives while effectively reducing false negatives.

As evidenced by the results in Table 6, our proposed method achieves approximately 1% higher precision across both datasets. This reflects its ability to reduce false positives. The inclusion of a person detector in our approach plays a critical role, validating handgun detections within a person’s bounding region and reducing false alarms.

Additionally, the recall values in Table 7 and Table 8 consistently match or surpass those reported in [10]. This demonstrates the robustness of our method in identifying concealed handguns, even under challenging conditions. Our conservative approach, where alarms are triggered if no person is detected but a handgun is identified, ensures that critical threats are not overlooked. This addresses a key limitation of false negatives, which could compromise security.

These results underline the effectiveness of our two-stage detection approach. The combination of a handgun detector and a person detector enhances accuracy while providing a balanced solution that prioritizes safety and reliability. Compared to [10], our method demonstrates a superior trade-off between precision and recall, making it highly suitable for real-world security applications where minimizing false positives and reducing false negatives are critical.

### 4.5. Demo

In addition to the study carried out, an Android app implementing the proposed method has been developed: CamoVision. It has been created using Android Studio and tested in a Huawei P30 Lite (Android 10). To test the method, only one model was used, the YOLOv8n, trained to detect two classes: “Handgun” and “Person”. The model was trained using the “UCLM Thermal Imaging Dataset”. The aim is to use only one model instead of the two architectures in order to perform better in embedded devices. The hyperparameters used to train the model are shown in Table 9.

**Figure 13:**
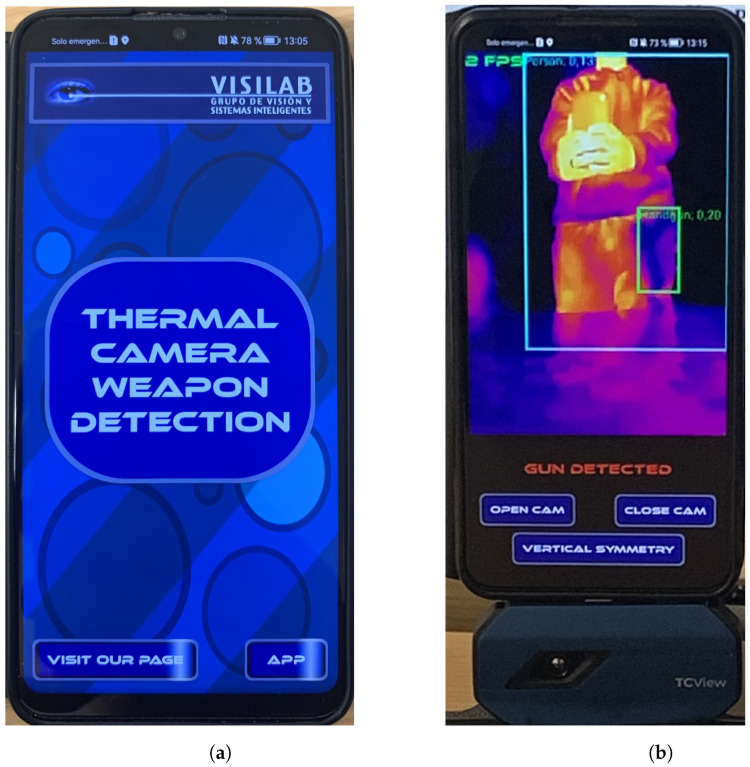
(**a**) CamoVision app menu. (**b**) CamoVision demo working with the TOPDON TC001 attached to the phone via USB bottom part of the image.

The code implements the proposed method considering only the case where the detection with the highest score for each class is selected. The app consists of two main screens (see Figure 13): a menu, where the user can click on a button that redirects him/her to our website (https://visilab.etsii.uclm.es/?page_id=1029, accessed on 5 October 2024), or a button to click to access the detector. This detector constitutes the second main screen of the app. It starts as a black screen with three buttons on it. One button is to open the thermal camera once it is attached via Universal Serial Bus (USB) to the smartphone; another one is to close the camera; the third one is to reflect the image over the vertical axis (depending on where the thermal camera is facing). Now, the thermal camera needs to be connected and, by clicking on the “open cam” button, permissions are requested, and, if conceded, the camera opens and the detector starts working.

**Figure 14:**
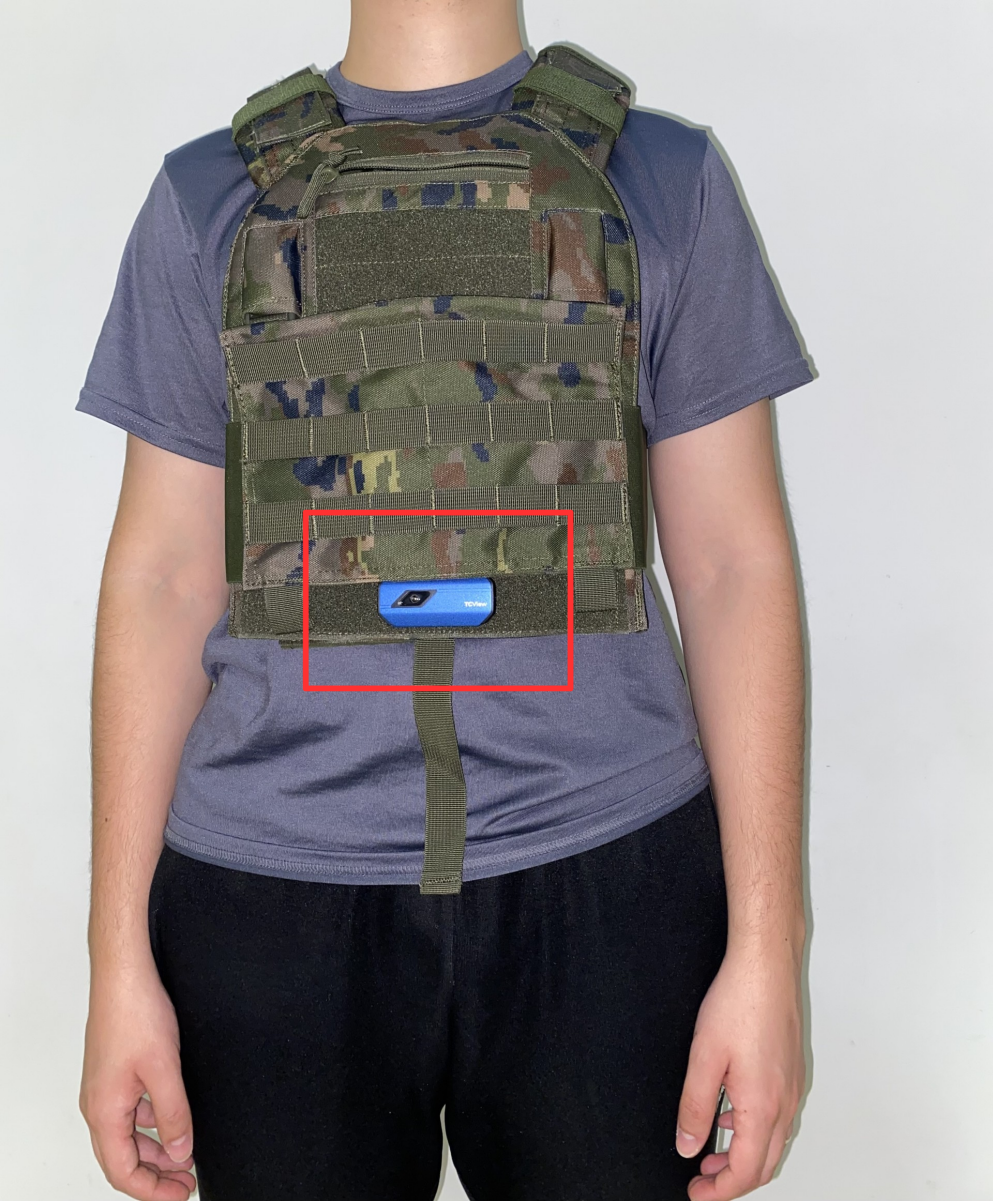
Example of how the CamoVision app would work for a policeman. Thanks to the small size of the TOPDON TC001 camera, it can be easily attached to the officer’s chest while moving naturally. The camera is highlighted with a red rectangle.

The app shows an alarm message whenever a concealed handgun is found within the image. Also, if four consecutive images contain a concealed handgun in them, an alarm sounds. We used a minimum confidence threshold of 0.1 for the detector, in order not to miss detections (then the one with the highest score is selected). The app runs at 2 FPS. Finally, an image showing how the camera could be placed in a real situation is shown in Figure 14.

## 5. Discussion

### 5.1. Strengths of the Proposed Method

The proposed method offers several strengths that make it an effective solution for concealed weapon detection. It employs a two-stage process involving handgun and person detection, which reduces both false positives and false negatives—key objectives for ensuring detection reliability. False positives are minimized by integrating a secondary person detection step that validates handgun detections within a person’s region, thus reducing unnecessary alarms. On the other hand, false negatives are minimized by not discarding handguns detected in frames where no person is identified by the detector. In such cases, it is preferable to allow for potential false positives rather than risk missing actual handguns, as the primary goal is to prioritize safety and prevent critical threats. This conservative approach enhances the system’s reliability, particularly in scenarios where detection errors could have serious consequences.

Another strength is the creation of the “UCLM Thermal Imaging Dataset”, which provides a valuable resource tailored for concealed handgun detection. This dataset supports further research and development, helping to refine and test detection algorithms under realistic conditions. Additionally, the method is integrated into the Android app CamoVision, demonstrating its practicality for real-world applications. The app supports real-time operation using a chest-mounted thermal camera, enabling hands-free usage and enhancing field usability (see Figure 14).

The method’s recall-oriented design prioritizes detecting handguns over avoiding false positives, which is crucial for security applications. Despite this focus, efforts are made to strike a balanced solution to maintain practical usability. Furthermore, the use of affordable thermal cameras and compatibility with smartphones makes the solution scalable and cost-effective, facilitating broader deployments in diverse environments.

### 5.2. Weaknesses of the Proposed Method

Despite its strengths, the proposed method also has some limitations. It depends heavily on the quality of the dataset, and while the “UCLM Thermal Imaging Dataset” was created for this study, it lacks diversity in environmental settings, lighting conditions, and subject demographics. These limitations may affect performance in more complex or unseen scenarios, as the system could struggle to generalize beyond the training data.

Another limitation is the speed of the method. The real-time implementation achieves only 2 FPS, which may be insufficient for high-traffic environments where faster processing and higher frame rates are required. Enhancing computational efficiency or leveraging sophisticated hardware could address this issue in future iterations.

The method is also currently focused on detecting a single person within the frame, limiting its effectiveness in crowded settings or scenarios involving multiple individuals. Incorporating multi-person detection capabilities could make the system more versatile and better suited for real-world applications involving larger groups.

Thermal cameras may face challenges in detecting objects concealed under thick or insulated clothing, as such materials can reduce the temperature contrast needed for detection. This limitation may hinder the method’s reliability in certain conditions and could be addressed by integrating multi-spectral imaging technologies or advanced preprocessing techniques.

Additionally, the method’s performance is influenced by confidence and IoU thresholds. During testing, low thresholds were used to maximize recall, but real-world deployments may require careful tuning to balance precision and recall, minimizing false positives while ensuring critical detections are not missed.

Finally, the architectures have been trained to detect only concealed handguns. Expanding the detection scope to include other concealed objects, such as knives or explosives, could significantly improve usability and versatility, broadening the range of applications for the proposed method.

### 5.3. Future Improvements and Conclusions

Several enhancements can be made to address the identified weaknesses and further improve the method’s performance.

- Expanding the dataset with more diverse scenarios, including variations in clothing, lighting, and environments, can improve generalization and adaptability. This would help the model better handle challenging and unseen situations, ensuring robustness across diverse settings.
- Incorporating multi-person detection capabilities will increase the system’s applicability in crowded spaces, enabling it to process more complex scenarios involving multiple individuals. Such an enhancement could make the method more practical for environments like airports, train stations, and large public gatherings.
- Improving the algorithm’s computational efficiency can significantly enhance its FPS, supporting faster real-time processing. This optimization would enable smoother operation in high-traffic areas where rapid decision-making is crucial.
- To address limitations in detecting objects under thick clothing, advanced thermal imaging techniques, such as multi-spectral imaging, can be integrated. These techniques can enhance detection performance by leveraging additional spectral data, making the system more reliable under challenging conditions.
- Adaptive thresholding, which dynamically adjusts detection parameters based on environmental conditions, can further balance precision and recall. This approach could ensure optimal performance even in varying lighting and thermal environments.
- Expanding the detection scope to include other concealed weapons or objects, such as knives and explosives, will enhance the system’s versatility and broaden its range of applications. This improvement would increase its utility in various security scenarios beyond handgun detection.
- Improving embedded system performance through hardware upgrades or algorithm optimizations can enhance usability, particularly for real-time applications. Faster hardware and more efficient algorithms would make the method more responsive and scalable.
- Finally, enhancing the Android app’s user interface by adding features such as cloud storage, remote monitoring, and integration with security networks can increase operational effectiveness. These upgrades would provide a more seamless experience for security personnel, enabling better data management and situational awareness.

Apart from the explained weaknesses and its possible future improvements, the proposed method represents a significant advancement in concealed weapon detection by combining thermal imaging and deep learning. It offers efficiency, portability, and accuracy, addressing many limitations of traditional detection methods. While the method demonstrates promising results (in terms of our aim), further improvements in datasets, algorithms, and capabilities can enhance its effectiveness. This research provides a foundation for developing scalable, real-world security solutions for non-invasive, real-time detection applications.

## Figures and Tables

**Table 1:** Summary of the dataset split and chosen hyperparameters to train the YOLOv3u handgun detector for the proposed method.

Architecture	Dataset	# Images	Classes	Train/Val/Test Images
**YOLOv3u**	UCLM Thermal Imaging Dataset	2402	“Handgun”	1441/239/722
	Concealed Pistol Detection Dataset	358	“Handgun”	214/35/109

**Table 2:** Table showing the main metrics extracted from testing the complete proposed method on the “UCLM Thermal Imaging Dataset” and “Concealed Pistol Detection Dataset” datasets. The mean and the standard deviation of the five runs considered for each dataset are shown; 0.0001 confidence score threshold for each architecture.

Dataset	Detections	Average Precision (IoU = 0.50:0.95, Area = All)	Standard Deviation
UCLM Thermal Imaging Dataset [16]	All	64.52%	0.0603
	Best score	64.30%	0.0606
Concealed Pistol Detection Dataset [11]	All	27.78%	0.0781
	Best score	27.70%	0.0843

**Table 3:** Table showing the main metrics extracted from ablation experiment (i.e., removing the person detector from the proposed method) on the “UCLM Thermal Imaging Dataset” and “Concealed Pistol Detection Dataset” datasets. The mean and the standard deviation of the five runs considered for each dataset. 0.0001 confidence score threshold for the handgun detector are shown.

Dataset	Detections	Average Precision (IoU = 0.50:0.95, Area = All)	Standard Deviation
UCLM Thermal Imaging Dataset [16]	All	64.38%	0.0596
	Best score	64.20%	0.0601
Concealed Pistol Detection Dataset [11]	All	27.57%	0.0761
	Best score	27.62%	0.0838

**Table 4:** Table showing classification metrics extracted from testing the proposed method on the “UCLM Thermal Imaging Dataset”. Only the detection with the highest score was considered per frame.

	Classification Metrics: UCLM Thermal Imaging Dataset			
Confidence Value	0.0001	0.1	0.5	0.75
**True Positives (TP)**	353	346	339	269
**False Positives (FP)**	252	42	15	4
**True Negatives (TN)**	117	327	354	365
**False Negatives (FN)**	0	7	14	84
**Accuracy (%)**	65.10	93.21	95.98	87.81
**Precision (%)**	58.35	89.18	95.76	98.53
**Recall (%)**	100.00	98.02	96.03	76.20
**F1-score**	0.74	0.93	0.96	0.86

**Table 5:** Table showing classification metrics extracted from testing the proposed method on the “Concealed Pistol Detection Dataset”. Only the detection with the highest score was considered per frame.

	Classification Metrics: Concealed Pistol Detection Dataset			
Confidence Value	0.0001	0.1	0.5	0.75
**True Positives (TP)**	52	45	7	0
**False Positives (FP)**	35	2	0	0
**True Negatives (TN)**	22	55	57	57
**False Negatives (FN)**	0	7	45	52
**Accuracy (%)**	67.89	91.74	58.72	52.29
**Precision (%)**	59.77	95.74	100.00	0
**Recall (%)**	100.00	86.57	13.46	0
**F1-score**	0.75	0.91	0.24	0

**Table 6:** Table showing the main metrics extracted from testing the complete proposed method along with the ones extracted from testing the method described in Veranyurt et al. [10]. The mean and the standard deviation of the five runs considered for each dataset are shown.

Method	Dataset	Detections	Average Precision (IoU = 0.50:0.95, Area = All)	Standard Deviation
**Proposed Method**	UCLM Thermal Imaging Dataset	All	64.52%	0.0603
		Best score	64.30%	0.0606
	Concealed Pistol Detection Dataset	All	27.78%	0.0781
		Best score	27.70%	0.0843
**Veranyurt et al. [10]**	UCLM Thermal Imaging Dataset	All	64.32%	0.0604
		Best score	64.10%	0.0601
	Concealed Pistol Detection Dataset	All	27.18%	0.0737
		Best score	27.32%	0.0806

**Table 7:** Table showing classification metrics comparing between the method described in Veranyurt et al. [10] and the proposed method. “UCLM Thermal Imaging Dataset”, only considering the detection with the highest score.

	Classification Metrics: UCLM Thermal Imaging Dataset							
Confidence Value	0.0001		0.1		0.5		0.75	
Method	Veranyurt et al. [10]	Proposed	Veranyurt et al. [10]	Proposed	Veranyurt et al. [10]	Proposed	Veranyurt et al. [10]	Proposed
**True Positives (TP)**	353	353	346	346	333	339	254	269
**False Positives (FP)**	343	252	6	42	0	15	0	4
**True Negatives (TN)**	26	117	363	327	369	354	369	365
**False Negatives (FN)**	0	0	7	7	20	14	99	84
**Accuracy (%)**	52.49	65.10	98.20	93.21	97.23	95.98	86.29	87.81
**Precision (%)**	50.72	58.35	98.30	89.18	100.00	95.76	100.00	98.53
**Recall (%)**	100.00	100.00	98.02	98.02	94.33	96.03	71.85	76.20
**F1-score**	0.67	0.74	0.98	0.93	0.97	0.96	0.84	0.86

**Table 8:** Table showing classification metrics comparing between the method described in Veranyurt et al. [10] and the proposed method. “Concealed Pistol Detection Dataset”, only considering the detection with the highest score.

	Classification Metrics: Concealed Pistol Detection Dataset							
Confidence Value	0.0001		0.1		0.5		0.75	
Method	Veranyurt et al. [10]	Proposed	Veranyurt et al. [10]	Proposed	Veranyurt et al. [10]	Proposed	Veranyurt et al. [10]	Proposed
**True Positives (TP)**	52	52	45	45	7	7	0	0
**False Positives (FP)**	57	35	2	2	0	0	0	0
**True Negatives (TN)**	0	22	55	55	57	57	57	57
**False Negatives (FN)**	0	0	7	7	45	45	52	52
**Accuracy (%)**	47.71	67.89	91.74	91.74	58.72	58.72	52.29	52.29
**Precision (%)**	47.71	59.77	95.74	95.74	100.00	100.00	0	0
**Recall (%)**	100.00	100.00	86.57	86.57	13.46	13.46	0	0
**F1-score**	0.65	0.75	0.91	0.91	0.24	0.24	0	0

**Table 9:** Summary of the dataset split and chosen hyperparameters to train the YOLOv8n detector (with two classes: “Handgun”, “Person”) used to test the proposed method in real-time through an Android app. “UCLM Thermal Imaging Dataset”.

Architecture	Dataset	# Images	Classes	Train/Val/Test Images	Epoch	Input Size	Optimizer	Learning Rate
**YOLOv8n**	Ours (UCLM Thermal Imaging Dataset)	12,006	“Handgun”, “Person”	10,805/1201/0	5	640 × 640	AdamW (0.9 momentum)	0.001667 (initial)

## Data Availability

The dataset created by the authors and used to test the methods can be found and downloaded at https://data.mendeley.com/datasets/b6rpgr6nrh/1 (accessed on 20 September 2024). We also used the Concealed Pistol Detection Dataset, which can be found at https://figshare.com/articles/dataset/Concealed_Pistol_Detection_Dataset/20105600?file=35965742 (accessed on 20 September 2024).
